# Which behavioral regulations predict physical activity and sedentary behavior in people with mental illness?

**DOI:** 10.1017/S0033291724001879

**Published:** 2024-11

**Authors:** Justin Chapman, Nicole Korman, Eva Malacova, Caroline Robertson, Urska Arnautovska, Dan Siskind, Shuichi Suetani, Brendon Stubbs, Davy Vancampfort

**Affiliations:** 1 Centre for Mental Health, Griffith University, Brisbane, QLD, Australia; 2 Addictions and Mental Health Service, Metro South Health, Brisbane, QLD, Australia; 3 QIMR Berghofer Medical Research Institute, Brisbane, QLD, Australia; 4 School of Medicine, The University of Queensland, Brisbane, QLD, Australia; 5 Queensland Centre for Mental Health Research, Brisbane, QLD, Australia; 6 Institute for Urban Indigenous Health, Windsor, Australia; 7 School of Medicine and Dentistry, Griffith University, Southport, QLD, Australia; 8 Department of Psychological Medicine, Institute of Psychiatry, Psychology and Neuroscience (IoPPN), King's College London, London, UK; 9 Department of Rehabilitation Sciences, KU Leuven, Leuven, Belgium

**Keywords:** mental health, physical activity, sedentary behaviour, motivation, behavioural regulation, self-determination, mental illness

## Abstract

**Background:**

Moderate-to-vigorous physical activity (MVPA) is beneficial for health, and reducing sedentary behavior (SB) is recommended in international guidelines. People with mental illnesses are at higher risk of preventable diseases than the general population, partly attributable to lower MVPA and higher SB. Self-determination theory provides a framework for understanding how motivation regulates behavior. This study aimed to evaluate the contribution of different forms of motivation for physical activity (amotivation, controlled, autonomous) to MVPA and SB in people with mental illnesses.

**Methods:**

Cross-sectional self-reported and accelerometer-derived MVPA and SB in people with a range of mental illnesses across four countries were pooled for analysis (Australia, Belgium, England, Uganda). Motivation for physical activity was measured using the Behavioural Regulation in Exercise Questionnaire (BREQ). Regression analyses were used to investigate the association of MVPA and SB with amotivation, controlled, autonomous motivations, controlling for mental health and demographic variables.

**Results:**

Autonomous motivation was associated with 31% higher self-reported MVPA, and amotivation and controlled motivation were associated with 18% and 11% lower self-reported MVPA, respectively (*n =* 654). In contrast, controlled motivation was positively associated with SB (*n =* 189). Having physical comorbidities or an alcohol use disorder was associated with lower MVPA (*n* = 318). Sub-analyses with accelerometer-derived MVPA and SB (*n =* 139 and *n* = 145) did not reveal any associations with motivational forms.

**Conclusions:**

Findings with an international sample support the universal relevance of motivation in promoting health-related behavior. Strategies for facilitating autonomous motivation should be utilized by health professionals seeking to support people with mental illnesses to become physically active.

## Introduction

Physical activity, defined as any bodily movement that requires energy expenditure, has diverse benefits for physical and mental health (Rhodes, Janssen, Bredin, Warburton, & Bauman, 2017). Regular moderate-to-vigorous physical activity (MVPA) can reduce premature all-cause mortality by up to 31%, and reduce the risk of developing chronic diseases such as cardiovascular disease and diabetes by 20–30% (Warburton, Charlesworth, Ivey, Nettlefold, & Bredin, 2010). Evidence on the relationship between MVPA and mental health benefits is also well established, with meta-analyses indicating moderate-to-large effect sizes for reducing depression and anxiety (Rebar et al., 2015), and for improving cognition, functioning, and mental health symptoms in people living with severe mental disorders such as schizophrenia (Firth, Cotter, Elliott, French, & Yung, 2015; Korman et al., 2023). World Health Organization guidelines for adults suggest at least 150–300 min per week of MVPA to achieve health-enhancing benefits (Bull et al., 2020). However, substantial benefit can be gained from comparatively small amounts of MVPA, and national guidelines have updated recommendations to include the statement ‘*some physical activity is better than none*’ (Bull et al., 2020). Similarly, the role of reducing sedentary behavior (SB) to improve health has also been highlighted in national guidelines (Bull et al., 2020), and systematic reviews report unfavorable associations with cognitive function, depression, function and disability, and quality of life (Saunders et al., 2020). Given that MVPA and SB are important independent, modifiable health-related behaviors, understanding their contributing factors for population groups that experience health disadvantage can inform the design of tailored interventions for these at-risk groups.

Globally, mental disorders are in the top 10 causes of disease burden (Collaborators, 2022), and are associated with physical co-morbidity (Plana-Ripoll et al., 2019). People with mental illnesses, particularly severe and persistent illnesses such as psychotic disorders, have a 10–20-year shorter life expectancy than the general population (Firth et al., 2019; Walker, McGee, & Druss, 2015). People with mental illness also have lower levels of MVPA and higher SB which contributes to this health disparity (Vancampfort et al., 2017). Supporting people with mental illness to improve these health-related behaviors is, therefore, a primary recommendation in landmark reviews such as the *Blueprint for protecting the physical health of people with mental illness* (Firth et al., 2019). Interventions to promote MVPA have demonstrated good adherence (withdrawal rates of 15–30% in people with depression or psychotic disorders) (Stubbs et al., 2016; Vancampfort et al., 2016c), and efficacy in reducing metabolic risk factors and improving symptoms of mental illnesses (Dauwan, Begemann, Heringa, & Sommer, 2015; Schuch et al., 2016); however, little is known about how to facilitate long-term healthy lifestyle behavior change in this group. Correlates that may influence adoption of MVPA in adults with mental illness include low self-efficacy, knowledge and beliefs, medical comorbidities, social isolation and loneliness, mental illness symptoms (depression, ‘negative’ symptoms of schizophrenia, hyperarousal, alcohol abuse), antipsychotic medications, having a diagnosis of schizophrenia (compared with depression or bipolar disorder), and demographic characteristics (e.g. age, sex, body mass index [BMI], low education, unemployment) (Suetani et al., 2016; Vancampfort et al., 2012; Vancampfort et al., 2013a; Vancampfort et al., 2015a, 2015c; Vancampfort et al., 2016b; Vancampfort et al., 2017). The literature on behavior change highlights the importance of motivation for increasing MVPA in the general population (Kwasnicka, Dombrowski, White, & Sniehotta, 2016; Schwarzer, Lippke, & Luszczynska, 2011; Teixeira, Carraça, Markland, Silva, & Ryan, 2012) and among people living with severe mental illness such as schizophrenia (Arnautovska et al., 2022).

Behavior is regulated by different forms of motivation which can be characterized by the degree of self-determination (Deci & Ryan, 1985). *Controlled motivations* relate to behaviors that are regulated by reward or punishment contingencies (external regulation) or by shame or guilt (introjected regulation) (Deci & Ryan, 2000; Ng et al., 2012). Examples of behaviors regulated by controlled motivation include someone who exercises because their doctor tells them to (external) or because they feel guilty if they do not exercise (introjected). *Autonomous motivations* relate to behaviors that are regulated by valuing the outcome (identified regulation), self-endorsing the activity as an aspect of identity (integrated regulation), or inherent satisfaction or enjoyment with the activity itself (intrinsic) (Deci & Ryan, 2008). Examples of behavior regulated by autonomous motivations include someone who exercises because they want to get fit (identified), because it is part of their identity, such as a sports player (integrated), or because they enjoy the sensation of exercising (intrinsic). *Amotivation*, on the other hand, describes avoidance of the activity. Autonomous motivations are higher in self-determination, and tend to translate into processes associated with more persistent behavior such as habit formation (Gardner & Lally, 2013). Despite the importance of motivation in health-related behaviors, a relatively small literature base is available on motivation for physical activity in people with mental illness.

Few studies have examined the relationship between different forms of motivation and MVPA in people with mental illness. One cross-sectional study found that intrinsic motivation for exercise was associated with 20-fold higher odds of being physically active (Sørensen, 2006). Analyses in this study did not explore potential differences by psychiatric diagnosis or control for health and demographic variables because of the small sample size (*n* *=* 109) (Sørensen, 2006). A review of cross-sectional studies involving people with mental illness included four studies that investigated self-determined motivation (Farholm & Sørensen, 2016). Of these, it was found that self-determined motivation was positively associated with self-reported MVPA (Sørensen, 2006; Vancampfort et al., 2013b; Vancampfort et al., 2015b) and stages of change (action and maintenance stages) (Vancampfort, Stubbs, Venigalla, & Probst, 2015d; Vancampfort et al., 2016a), and inversely associated with negative affect (e.g. distress, irritability, nervousness) (Vancampfort et al., 2015b). None of these studies controlled for other forms of motivation or health and demographic variables, or explored the association of different forms of motivation with SB. Understanding the relationship between different forms of motivation with MVPA and SB in people with mental illness can inform intervention strategies to improve health outcomes for this group.

The primary aim of this study was to determine whether different forms of motivation for physical activity are associated with MVPA in a diverse sample of people living with mental illnesses. A secondary aim was to investigate the relationships between different forms of motivation for SB. To address these aims, regression models were used to predict both self-reported and accelerometer-derived MVPA and SB. Hypotheses were that autonomous motivation would predict MVPA level, and that amotivation, but not controlled or autonomous motivation, would predict SB.

## Methods

Data from four countries and 10 different studies that were either cross-sectional designs or baseline measures from intervention studies were pooled for analyses (total *n* *=* 767; Australia = 408; Belgium = 221; England = 40; Uganda = 98). This study was carried out following the Declaration of Helsinki guidelines for human research. All participants gave their written informed consent. Ethical approval was obtained from QIMR Berghofer Medical Research Institute's Human Research Ethics Committee to combine data from international collaborators (P2398). The characteristics of these studies and corresponding participant samples are provided in online Supplementary Table 1; the measures used in these studies are outlined below.

### Measures

#### Exercise motivation

The *Behavioural Regulation in Exercise Questionnaire* is designed to measure the behavioral regulations for physical activity and exercise informed by self-determination theory (Markland & Tobin, 2004). Example questions include: ‘*It's important to me to exercise regularly*’, and ‘*I don't see why I should have to exercise*’; scoring is on a 5-point scale ranging from 0 (‘Not true for me’) to 4 (‘Very true to me’) (Markland & Tobin, 2004), and questionnaire items relating to each of the behavioral regulation subscales (amotivation, external, introjected, identified, integrated and intrinsic regulations) are averaged to obtain a score for each behavioral regulation (Cavicchiolo et al., 2022). These subscales were then collapsed into three clinically relevant categories: *amotivation*, *controlled motivation* (average of external and introjected regulations) and *autonomous motivation* (average of identified, integrated and intrinsic regulations) (Ng et al., 2012). The relative autonomy index (RAI) was also calculated as the weighted sum of subscales resulting in a continuous score representing motivation across the spectrum of self-determination (Cavicchiolo et al., 2022); RAI was used only for visualizing data.

#### Physical activity and sedentary behavior

The *International Physical Activity Questionnaire* – *short form* (IPAQ) consists of seven items to assess weekly frequency and average daily duration of activity and SB in the previous 7 days (Craig et al., 2003). Activities assessed include vigorous activities (e.g. heavy lifting, digging, aerobics, fast cycling), moderate activities (e.g. carrying light loads, cycling, tennis), and walking (at work or home, and for traveling, recreation, sport, exercise, or leisure). The IPAQ has a test–retest reliability of 0.68 and validity coefficient of 0.37 when compared with accelerometer-derived estimates of MVPA for people with schizophrenia (Faulkner, Cohn, & Remington, 2006).

The *2-item Physical Activity Questionnaire* (2PAQ) was adapted from the Physical Activity as a Vital Sign (PAVS) questions (Greenwood, Joy, & Stanford, 2010) which ask about activity at least 30 min duration completed in the previous week. The PAVS has been adapted in studies with people with mental illness to focus on an average week (instead of previous week), and on MVPA of any duration (rather than a minimum duration) (Vancampfort et al., 2016d). The 2PAQ was adapted to ask specifically about *purposeful exercise* rather than MVPA: (i) *On average, how many days per week do you engage in purposeful exercise* (*not just incidental*)*?*; and (ii) *On these days, on average, how much time do you spend doing this exercise?*. The number of days was multiplied by the duration to estimate weekly MVPA.

The *Simple Physical Activity Questionnaire* (SIMPAQ) is a 5-item interviewer-administered questionnaire to assess physical activity and SB in the previous 7 days. Questions ask about time spent in bed for sleep, sitting or lying down, walking (for exercise, recreation, or travel), other exercise or sport (e.g. running, swimming, cycling, sport), and incidental activities (for work, gardening, household chores). The SIMPAQ has test–retest reliability of 0.69 for SB and 0.76 for walking and exercise; self-reported MVPA has a correlation of 0.25 with accelerometer-derived MVPA in a diverse sample of people with mental illnesses (Rosenbaum et al., 2020). Self-reported SB was obtained from four studies in units of minutes/day.

*Accelerometry:* Accelerometer-derived MVPA and SB were obtained from four studies that used the GENEActiv wrist-worn monitor (GENEActiv, Activinsights Ltd, Kimbolton, UK) as a baseline assessment of activity. The monitors measure motion-related and gravitational acceleration using a triaxial microelectromechanical systems accelerometer, light exposure using a photodiode, and temperature using a thermistor. Data were analyzed with R-package GGIR version 2.1–0 R (https://cran.r-project.org/web/packages/GGIR/) (Migueles, Rowlands, Huber, Sabia, & van Hees, 2019); non-wear time was defined using GGIR defaults as 60 min of inactivity based on the value range of each axis calculated for 60 min windows with 15 min sliding windows, invalid data were imputed by the average at similar times of different days of the monitoring period (Van Hees et al., 2013). Participants were asked to wear accelerometers for 7 consecutive days; accelerometer data were considered valid if they had at least 10 h of wear time. The number of valid accelerometer days for participants was mean = 4.6 (s.d. = 2.0) and the non-wear time was mean = 0.4% (s.d. = 1.5%) of waking hours.

#### Mental health

The *Kessler-6 scale of psychological distress* (K6) consists of six items to assess general psychological distress (*nervous*, *hopeless*, *restless or fidgety*, *so sad nothing could cheer you up*, *sense of effort*, *worthless*) experienced in the past month using a 5-point scale. The K6 has high internal consistency and reliability (Cronbach's *α* = 0.89). Scores range from 6 to 30, with a total score over 15 indicating high distress in adults (Kessler et al., 2003). The high distress threshold has a total classification accuracy of 0.92 for screening for severe mental illness in the general population (Kessler et al., 2003).

The *Brief Psychiatric Rating Scale* (BPRS) is a semi-structured interview designed to assess psychiatric symptoms in people with psychotic disorders (Crippa, Sanches, Hallak, Loureiro, & Zuardi, 2001). The interview consists of 18 items which are rated on a 7-point scale; BPRS scores were dichotomized as score > 41 corresponding with moderate-to-severe symptoms as rated by the Clinical Global Impressions Severity scale (Leucht et al., 2005).

*Psychiatric diagnosis* was collected from medical chart review in nine studies (*n* *=* 528) and was self-reported in one study (*n* *=* 239). Primary diagnosis was categorized as follows: psychotic disorder (schizophrenia, schizoaffective disorder), affective disorder (depression, bipolar disorder), substance use disorder (alcohol and/or drug), other (total *n* *=* 24, comprising: borderline personality disorder, *n* *=* 6; anxiety disorder, post-traumatic stress disorder, *n* *=* 12; eating disorder, *n* *=* 1; attention deficit/hyperactivity disorder, adjustment disorder, autism spectrum disorder, *n* *=* 5).

#### Physical health and comorbidities

The Adult Pre-exercise Participation System (APSS) medical screening is administered by a health professional to assess medical history and suitability for exercise. Multimorbidity was dichotomized using co-morbidities identified using the medical screening questionnaire: ‘0 or 1 comorbidity’, and ‘2 or more comorbidities’. BMI was left as a continuous variable; smoking status was dichotomous (‘smoker’ *v.* ‘non-smoker’).

### Statistical analyses

Because of the high number of participants reporting zero minutes/week of MVPA and the variance being greater than the mean (over-dispersion), a zero inflated negative binomial regression (ZINB) was used to evaluate the effect of amotivation, controlled motivation, and autonomous motivation on self-reported MVPA, with ‘study’ as a clustering variable to ensure robust standard errors and reliable inferences. Because age, sex, and BMI are well-established correlates of MVPA (Suetani et al., 2016; Vancampfort et al., 2012; Vancampfort et al., 2013a; Vancampfort et al., 2015a, 2015c; Vancampfort et al., 2016b; Vancampfort et al., 2017), they were included as covariates along with the questionnaire type and psychiatric diagnosis.

Additional subgroup analyses were conducted to further investigate potential confounders: (i) an additional three variables were included in ZINB analyses for self-reported MVPA (mental distress, multimorbidity, and smoking status); and (ii) accelerometer-derived MVPA was investigated using mixed-effects regression with random intercepts using fewer variables because of the smaller sample size (only including motivation variables, controlling for age, sex, and BMI and using study as a clustering variable). Because of the smaller sample size, these same variables were also used for secondary analyses, in which mixed-effects regression models with random intercept were used to predict self-reported and accelerometer-derived SB.

The variables were normally distributed except for accelerometer-derived MVPA which was approximately log-normally distributed, so a log transformation was used for this sub-analysis and results were back-transformed. Homogeneity of variance was confirmed by examining plots of residuals *v.* fitted values, and using the White's test to check for heteroscedasticity. The variance inflation factor was examined to confirm that the variables did not violate multicollinearity. Sample size guidelines for regression analyses vary; however, using the 20 ‘events per variable’ guideline (Austin & Steyerberg, 2017), sample sizes were sufficient for all analyses (*n* > 120 needed for accelerometer sub-analyses; *n* > 260 needed for sub-analyses with self-reported MVPA). Significance was set at *p* < 0.05; all analyses were conducted in STATA (StataCorp. 2021. Stata Statistical Software: Release 17.0. College Station, TX, USA: StataCorp LLC.).

## Results

**Figure 1.**
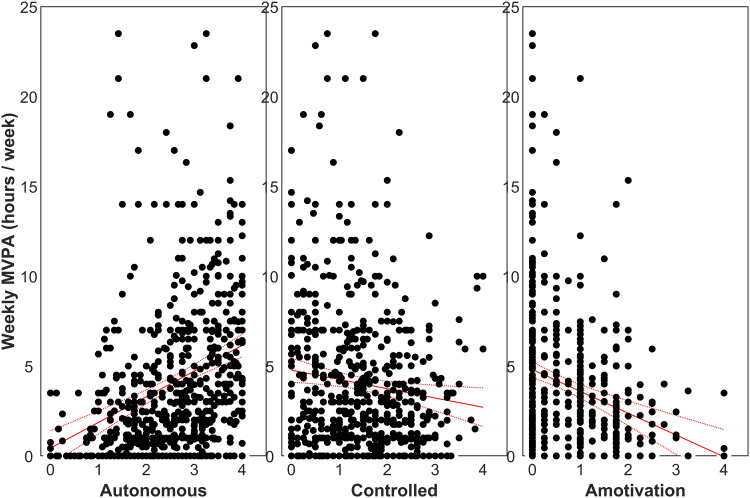
Scatter plots of self-reported moderate-to-vigorous physical activity (MVPA; *n* = 654) against autonomous motivation (left), controlled motivation (middle), and amotivation (right). MVPA was correlated with autonomous motivation (*r* *=* 0.282, *p* < 0.001), controlled motivation (*r* *=* *−*0.127, *p* *=* 0.001), and amotivation (*r* *=* *−*0.221, *p* < 0.001). Regression lines are plotted with the standard error of the mean.

**Figure 2.**
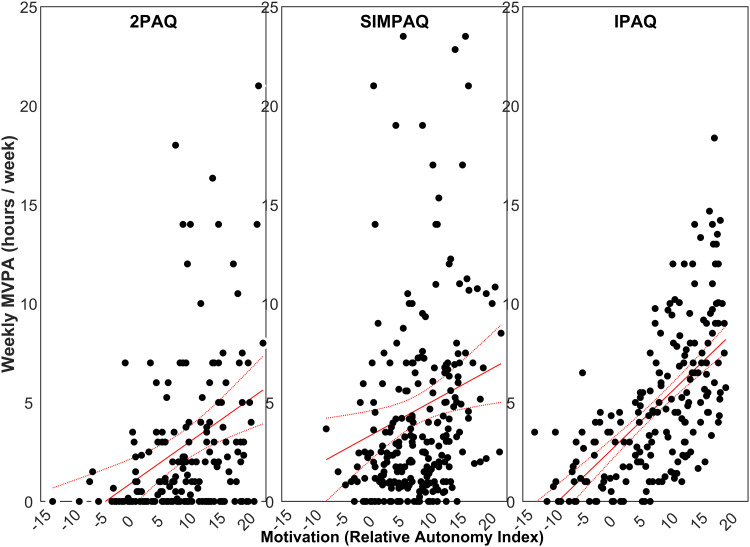
Scatter plots of self-reported moderate-to-vigorous physical activity (MVPA) against motivation scored as a relative autonomy index (RAI) for the three questionnaires used in the included studies: *2-item Physical Activity Questionnaire* (2PAQ: left), *Simple Physical Activity Questionnaire* (SIMPAQ: middle), and *International Physical Activity Questionnaire – short form* (IPAQ: right). MVPA was correlated with RAI for the 2PAQ (*r* *=* 0.295, *p* < 0.001; *n* *=* 186), SIMPAQ (*r* *=* 0.179, *p* *=* 0.004; *n* *=* 247), and IPAQ (*r* *=* 0.630, *p* < 0.001; *n* *=* 221). The regression of self-reported MVPA against controlled RAI is plotted with the standard error of the mean for each questionnaire.

A total of 654 participants had complete data for the main analyses. Participants had a mean age of 38 years, most were overweight (58% with BMI > 25 kg/m^2^), the most common diagnostic category was affective disorders (46%) closely followed by psychotic disorders (42%), and there were approximately equal numbers of males and females (Table 1). The ZINB regression model (Table 2) indicated significant relationships between MVPA and all motivational forms: each unit increase in autonomous motivation was associated with 31% increase in MVPA (RR 1.31; 95% CI 1.13–1.53; *p* < 0.001), whereas amotivation and controlled motivation were associated with 18% and 11% decreases in MVPA (RR 0.82; 95% CI 0.69–0.97; *p* = 0.022; and RR 0.89; 95% CI 0.81–0.97; *p* = 0.008), respectively. The ‘zero component’ of the ZINB regression also indicated strong associations between reporting zero minutes/week of MVPA and amotivation (OR 1.63; 95% CI 1.23–2.14; *p* = 0.001) and autonomous motivation (OR 0.58; 95% CI 0.46–0.73; *p* < 0.001). Plots illustrating the relationship between MVPA and motivational forms are shown in Fig. 1. The questionnaire used was also strongly associated with MVPA: compared with the 2PAQ, the SIMPAQ was associated with 56% higher MVPA (RR 1.56; 95% CI 1.18–2.04; *p* = 0.002) and the IPAQ was associated with 31% higher MVPA (RR 1.31; 95% CI 1.16–1.47; *p* < 0.001). SIMPAQ and IPAQ questionnaires were also associated with lower odds of reporting zero minutes/week of MVPA (SIMPAQ: OR 0.13; 95% CI 1.23–2.14; *p* < 0.001; and IPAQ: OR 0.09; 95% CI 0.07–0.11; *p* < 0.001). Figure 2 illustrates different patterns of responses for each questionnaire; the proportions of participants reporting zero minutes using the 2PAQ compared with the SIMPAQ and IPAQ were 43% *v.* 10% and 9%, respectively. When considering psychiatric diagnosis, people with alcohol and/or drug use disorders had lower MVPA (RR 0.61; 95% CI 0.40–0.93; *p* *=* 0.021) than those with psychotic disorders. Age, sex, and BMI were not significantly associated with MVPA.

**Table 1.** Participant characteristics for primary and secondary analyses

Primary analysis (*n* = 654)		
	Mean	(s.d.)
*Motivation*		
Amotivation (range = 0–4)	0.6	(0.8)
Controlled motivation (range = 0–4)	1.4	(0.9)
Autonomous motivation (range = 0–4)	2.5	(0.9)
Age (range = 16–80 years)	38.0	(13.2)
BMI (range = 16.2–65.4 kg/m^2^)	28.2	(7.8)
Self-reported MVPA (range = 0–1680 min/week)	238.5	(271.1)
Accelerometer-derived MVPA (*n* = 145; range = 10.6–197.2 min/day)	68.3	(38.7)
	*n*	(*%*)
*Physical activity questionnaire*		
2PAQ	186	(28.4)
SIMPAQ	247	(37.8)
IPAQ	221	(33.8)
*Psychiatric diagnosis*		
Psychotic disorder	278	(42.5)
Affective disorder	303	(46.3)
Alcohol and/or drug use disorder	54	(8.3)
Other	19	(2.9)
*Sex*		
Male	337	(51.5)
Female	317	(48.5)
*Country*		
Australia	337	(51.5)
Belgium	221	(33.8)
Uganda	96	(14.7)
**Secondary analysis (*n* = 189)**		
	Mean	(s.d.)
Self-reported SB (range = 0–930 min/day)	439.2	(202.9)
Accelerometer-derived SB (*n* = 145; range = 161–1018 min/day)	554.1	(138.5)
*Motivation*		
Amotivation (range = 0–3)	0.5	(0.7)
Controlled motivation (range = 0–3.9)	1.4	(1.0)
Autonomous motivation (range = 0–4)	2.4	(0.9)
Age (range = 19–64 years)	35.4	(10.6)
BMI (range = 17.2–56.3 kg/m^2^)	31.5	(7.4)
	*n*	(*%*)
*Sex*		
Male	122	(64.6)
Female	67	(35.4)
*Country*		
Australia	151	(79.9)
England	38	(20.1)

MVPA, moderate-to-vigorous physical activity; SB, sedentary behavior; BMI, body mass index; 2PAQ, 2-item Physical Activity Questionnaire; SIMPAQ, Simple Physical Activity Questionnaire; IPAQ, International Physical Activity Questionnaire – short form.

**Table 2.** Regression statistics to predict moderate-to-vigorous physical activity (MVPA) and sedentary behavior (SB)

Dependent variable	Independent variables	Effect	95% Confidence intervals		Significance
*Primary analyses*					
Self-reported MVPA (*n* = 654)	*ZINB count component*	*Risk ratio*	*Lower*	*Upper*	*p* *>* *|z|*
	Amotivation	0.82	0.69	0.97	0.022*
	Controlled motivation	0.89	0.81	0.97	0.008*
	Autonomous motivation	1.31	1.13	1.53	<0.001**
	Age (10-year units)	0.96	0.93	1.00	0.058
	*Sex* (*ref: male*)				
	Female	0.92	0.73	1.15	0.436
	BMI (10 kg/m^2^ units)	0.98	0.88	1.09	0.708
	*Questionnaire* (*ref: 2PAQ*)				
	SIMPAQ	1.56	1.18	2.04	0.002**
	IPAQ	1.31	1.16	1.47	<0.001**
	*Diagnosis* (*ref: psychotic disorder*)				
	Affective disorder	0.98	0.87	1.11	0.775
	Alcohol and/or drug use disorder	0.61	0.40	0.93	0.021*
	Other diagnoses	1.04	0.85	1.26	0.706
	***ZINB zero component***	*Odds ratio*	*Lower*	*Upper*	*p* *>* *|z|*
	Amotivation	1.63	1.23	2.14	0.001**
	Autonomous motivation	0.58	0.46	0.73	<0.001**
	*Questionnaire* (*ref: 2PAQ*)				
	SIMPAQ	0.13	0.06	0.26	<0.001**
	IPAQ	0.09	0.07	0.11	<0.001**
*Sub-analysis* Self-reported MVPA (*n* = 318)	***ZINB count component***	*Risk ratio*	*Lower*	*Upper*	*p* *>* *|z|*
	Amotivation	0.99	0.69	1.40	0.940
	Controlled motivation	0.90	0.77	1.06	0.217
	Autonomous motivation	1.28	0.90	1.82	0.163
	Age (10-year units)	0.96	0.88	1.05	0.397
	*Sex* (*ref: male*)				
	Female	0.94	0.69	1.29	0.710
	BMI (10 kg/m^2^ units)	0.89	0.78	1.00	0.057
	*Questionnaire* (*Ref: 2PAQ*)				
	SIMPAQ	1.64	0.94	2.87	0.081
	*Diagnosis* (*ref: psychotic disorder*)				
	Affective disorder	0.85	0.54	1.34	0.479
	Alcohol and/or drug use disorder	0.37	0.17	0.81	0.012*
	Other diagnoses	0.84	0.53	1.33	0.449
	*Smoking status* (*ref: non-smoker*)				
	Smoker	1.16	0.92	1.45	0.206
	*Mental health* (*ref: low distress*)				
	High distress	1.14	0.97	1.34	0.107
	*Physical comorbidity* (*ref: no comorbidity*)				
	Two or more	0.77	0.70	0.84	<0.001**
	***ZINB zero component***	*Odds ratio*	*Lower*	*Upper*	*p* *>* *|z|*
	Autonomous motivation	0.63	0.58	0.67	<0.001**
	*Questionnaire* (*ref: 2PAQ*)				
	SIMPAQ	0.08	0.06	0.11	<0.001**
	*Physical comorbidity* (*ref: no comorbidity*)				
	Two or more	1.59	1.20	2.12	0.001**
*Sub-analysis* accelerometer-derived MVPA (*n* *=* *139*)	***Mixed-effects regression***	*Coefficient*	*Lower*	*Upper*	*p* *>* *|z|*
	Amotivation	0.98	0.84	1.16	0.842
	Controlled motivation	0.93	0.83	1.03	0.165
	Autonomous motivation	1.03	0.90	1.18	0.681
	Age (10-year units)	0.83	0.75	0.93	0.001**
	*Sex* (*ref: male*)				
	Female	1.05	0.85	1.29	0.658
	BMI (10 kg/m^2^ units)	0.94	0.82	1.08	0.397
***Secondary analyses***					
*Self-reported SB* (*n* *=* *189*)	***Mixed-effects regression***	*Coefficient*	*Lower*	*Upper*	*p* *>* *|z|*
	Amotivation	−24.81	−68.79	19.18	0.269
	Controlled motivation	65.48	34.54	96.42	<0.001**
	Autonomous motivation	−26.08	−61.64	9.47	0.151
	Age (10-year units)	15.85	−11.38	43.07	0.254
	*Sex* (*ref: male*)				
	Female	−38.02	−96.46	20.41	0.202
	BMI (10 kg/m^2^ units)	−25.88	−64.12	12.36	0.185
*Sub-analysis* accelerometer-derived SB (*n* *=* 145)	***Mixed-effects regression***	*Coefficient*	*Lower*	*Upper*	*p* *>* *|z|*
	Amotivation	1.61	−28.53	31.74	0.917
	Controlled motivation	−3.15	−23.57	17.27	0.762
	Autonomous motivation	−0.36	−25.45	24.73	0.977
	Age (10-year units)	16.31	−3.50	36.12	0.106
	*Sex* (*ref: male*)				
	Female	15.37	−23.61	54.34	0.440
	BMI (10 kg/m^2^ units)	−6.26	−32.09	19.56	0.635

*Note*: All analyses used ‘study’ as the clustering variable. ZINB, zero-inflated negative binomial regression; MVPA, moderate-to-vigorous physical activity in minutes/week; BMI: body mass index; 2PAQ: 2-item Physical Activity Questionnaire; SIMPAQ: Simple Physical Activity Questionnaire; IPAQ: International Physical Activity Questionnaire – short form. *p < 0.05, **p < 0.005.

When also accounting for mental distress, smoking status, and physical comorbidity in the ZINB regression model (*n* *=* 318; Table 2), the inverse association between MVPA and having an alcohol and/or drug use disorder remained significant (RR 0.37; 95% CI 0.17–0.81; *p* = 0.012). However, associations with motivational forms were no longer observed for MVPA as a continuous variable, and autonomous motivation was only associated with lower odds of reporting zero minutes/week of MVPA in the logistic part of the model (OR 0.63; 95% CI 0.58–0.67; *p* < 0.001). Multimorbidity was additionally inversely associated with MVPA (RR 0.77; 95% CI 0.70–0.84; *p* < 0.001). When investigating accelerometer-derived MVPA in sub-analyses, self-reported and accelerometer-derived estimates of MVPA were correlated (online Supplementary Fig. 1). However, only age was inversely associated with accelerometer-derived MVPA in the mixed-effects regression model, with each 10-year increase relating to a 17% decrease in MVPA (*r* = 0.83, 95% CI 0.75–0.93, *p* = 0.001).

**Figure 3.**
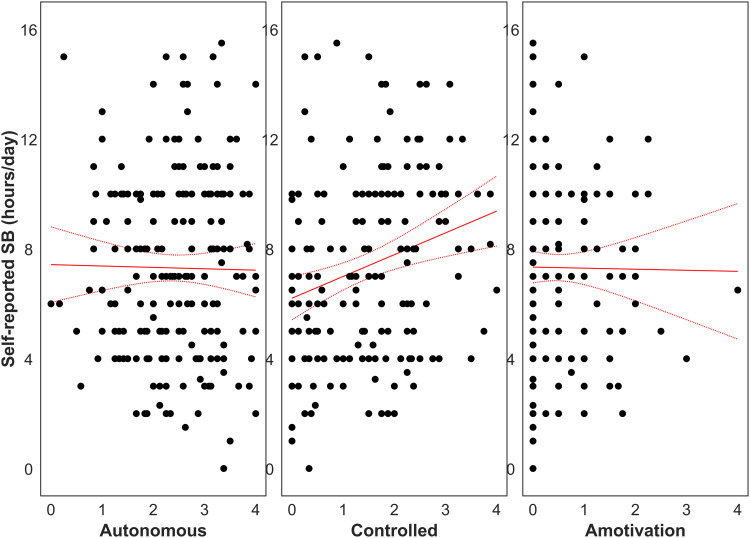
Self-reported sedentary behavior (SB; *n* *=* 189) for autonomous motivation (left), controlled motivation (middle), and amotivation (right). SB was correlated with controlled motivation (*r* *=* 0.24, *p* < 0.001), but not autonomous motivation (*p* *=* 0.85) or amotivation (*p* *=* 0.91). Regression lines are plotted with the standard error of the mean.

Secondary analyses included 189 participants with self-reported SB and 145 participants with accelerometer-derived SB. The mixed-effects regression models only revealed a significant association between controlled motivation for MVPA and self-reported SB (*r* *=* 65.48, 95% CI 34.54–96.42, *p* < 0.001; Fig. 3). While accelerometer-derived SB was correlated with self-reported values (online Supplementary Fig. 2), no associations were observed between SB and the explanatory variables investigated.

## Discussion

The aim of this study was to assess the relationship between different forms of self-determined motivation with MVPA and SB in people with mental illnesses. Consistent with our hypothesis, autonomous motivation was strongly associated with MVPA in people with mental illnesses: each unit increase of autonomous motivation for MVPA was associated with 31% higher MVPA, and about half the odds of reporting zero minutes/week of MVPA (OR = 0.58). Subgroup analyses investigating an expanded set of variables including mental distress, smoking status, and physical comorbidities confirmed the relationship between autonomous motivation and lower odds of reporting zero minutes/week of MVPA (OR = 0.63); however, the association with non-zero MVPA data was no longer evident. Both amotivation and controlled motivations were associated with lower MVPA, with each unit increase being related to 18% and 11% lower MVPA, respectively. Controlled motivation was additionally associated with self-reported SB (*r* = 65.48).

Studies with general population samples have similarly indicated that more autonomous forms of motivation are associated with MVPA; however, they have predominantly investigated a limited set of motivational types, or collapsed motivation into a composite score (RAI) (Teixeira et al., 2012). We investigated clinically relevant forms of amotivation, controlled and autonomous motivations to provide more specific recommendations about their relative contributions. Our findings confirm previous research conducted with smaller samples of people with mental illnesses (Costa et al., 2018; Vancampfort et al., 2013b; Vancampfort et al., 2015d; Vancampfort et al., 2016a). The central role of autonomous motivation is pertinent to health professionals seeking to help people with mental health issues to improve MVPA. While not everyone benefits from a high degree of autonomous motivation for exercise, motivations can become internalized over time with the right support. Autonomous motivation arises from personal identification with, and enjoyment of, the behavior and its benefits, which can be facilitated in environments that satisfy three psychological ‘needs’, i.e. the need for autonomy, competence, and relatedness (Deci & Ryan, 2000). It is therefore essential that health professionals understand how physical activity integrates with one's identity, life context, and personal preferences and abilities in an empowering person-centered way (Costa et al., 2018). Strategies that include minimizing pressure, promoting rewards, adopting supportive language and positive feedback, and promoting group-based activities with people experiencing similar challenges in a welcoming environment are related to program attendance and motivation (Arnautovska et al., 2022; Seymour et al., 2021; Whybird et al., 2020). This has been shown in an ecologically valid, community-based exercise program which resulted in higher autonomous motivation in those with mental illnesses (Seymour et al., 2021). Conversely, those participating in an exercise program in a more highly controlled setting showed reduced self-determination (Korman et al., 2020; Korman et al., 2018); however, these studies were with people with schizophrenia, who experience motivational difficulties because of cognitive changes associated with severe mental illness (Arnautovska et al., 2022). In consideration of the neurobiological processes occurring within schizophrenia that can impact uptake of MVPA, Arnautovska et al. (2022) highlighted that an individual's prediction of the value of rewards associated with an activity, in particular ‘salience’, is also part of the motivational process impacted in people with severe mental illnesses. Reduced prediction of rewards may show in the underestimation of potential positive experiences and benefits of MVPA (Arnautovska et al., 2022).

Controlled motivation for MVPA was associated with SB and inversely associated with MVPA. This indicates that prescriptive, non-consultative techniques that result in controlled motivations for MVPA may not have the desired effect for people with mental illnesses. Such approaches in the absence of individualized support may only serve to lower self-efficacy or increase feelings of guilt if they are unable to overcome their personal barriers to physical activity. For people with severe mental illnesses, such approaches may also be reminiscent of coercive or authoritarian relationships experienced in other settings (Hughes, Hayward, & Finlay, 2009), which may lead to avoidance of the behavior. Few studies have investigated associations between motivation and SB: one study investigated motivation for sedentary activities (rather than motivation for MVPA as assessed in our study), reporting that autonomous and controlled motivations for SB were associated with SB in recreational and work contexts, respectively (Gaston, De Jesus, Markland, & Prapavessis, 2016). Main factors associated with SB identified in general population samples include self-efficacy, social participation, social support, loneliness, anxiety, and depression (Prince, Reed, McFetridge, Tremblay, & Reid, 2017), highlighting the need for a supportive environment to improve relationships and self-perceptions which are likely to be poor in those with mental illness. Age (RR = 0.83) and physical comorbidities (RR = 0.77; OR = 1.59) were associated with lower MVPA, highlighting the importance of encouraging activity throughout aging and for preventative purposes before the development of physical health conditions form additional barriers to becoming physically active. While having an alcohol and/or drug use disorder was associated with lower MVPA, this sample was predominantly inpatients in Uganda, so this may have been due to confounding environmental influences rather than associations with diagnosis.

This study is robust in that we had a relatively large international sample of people with a range of mental illness from diverse settings. Data were pooled from different studies across Australia, England, Uganda, and Belgium, indicating that the relative contributions of different forms of motivation are important for people with mental illness across diverse cultural and socioeconomic settings. The specific self-report questionnaire used was associated with MVPA, which is intuitive given that different questionnaires have different validity, and one of the questionnaires only asked about purposeful exercise which may influence self-reporting because of different perceptions of what constitutes exercise among participants. Self-report data were used for the main analyses, which are susceptible to reporting errors such as recall and social desirability bias (Firth et al., 2018). Accelerometry overcomes reporting limitations by algorithmically estimating MVPA and SB from bodily acceleration. Although self-report and accelerometer-derived measures of MVPA and SB were correlated, the associations with motivation were not consistent in subgroup analyses, which may be because of the smaller sample size and differences in how MVPA and SB are measured. Accelerometers detect small changes in movement which may not be associated with motivation for activity, whereas self-report questionnaires asked about the type and intensity of activity, which may explain why associations were only found between motivation and MVPA for self-reported estimates. This study only assessed cross-sectional associations which do not imply causation. Future studies should assess whether changes in motivation are related to changes in MVPA behavior and compare different behavioral interventions in this group. These findings are pertinent to informing approaches of physicians and health professionals seeking to facilitate health-related behavior change to improve wellbeing and prevent and/or manage deteriorating health in people with mental illness.

Self-determined motivation plays an important role in human behavior. Autonomous motivation for physical activity was associated with higher level of MVPA in people with a variety of diagnosed mental illnesses, and controlled motivation was associated with higher SB. People with a psychotic illness or substance use disorder, multiple physical comorbidities, and higher age may need additional support from family and carers and other health professionals to support adoption and maintenance of an active lifestyle. Strategies to increase autonomous motivation should be used to support behavior change for people with mental illness to improve wellbeing and prevent physical diseases.

## Supporting information

Chapman et al. supplementary material Chapman et al. supplementary material

## Supplementary material

The supplementary material for this article can be found at https://doi.org/10.1017/S0033291724001879.
