# Supplementary material for: Which behavioral regulations predict physical activity and sedentary behavior in people with mental illness?
Source: Psychol Med. 2024 Nov 21;54(15):4129–39. doi: 10.1017/S0033291724001879 (PMC11650172; doi:10.1017/S0033291724001879)
Supplement: Chapman et al. supplementary material [file S0033291724001879sup001.docx]

| **Supplementary Table 1: Description of studies and cross-sectional data from participant samples (total n=767)** | | | | | | |
| --- | --- | --- | --- | --- | --- | --- |
| *Study* | *Sample size* | *Measures* | | | | *Details* |
|  |  | *Questionnaire* | *Sedentary behaviour* | *Exercise motivation* | *Mental/ physical health* |  |
| Vancampfort et al^1^ | 56 | IPAQ | - | BREQ-2 | *-* | *Country*: Belgium  *Diagnosis*: First Episode Psychosis (n=56)  *Sex*: Female (n=20); Male (n=36)  *Demographics* *(males and females respectively)*  *Age*: M=25.0 (SD=3.7) and M=23.7 (SD=3.9) years  *BMI*: M=23.3 (SD=4.1) and 24.4 (SD=4.1) kg/m^2^ |
| Vancampfort et al^2,3^ | 165 | IPAQ | - | BREQ-2 | *-* | *Country*: Belgium  *Diagnoses*: Major depressive disorder (n=96); Bipolar disorder (n=69)  *Sex*: Female (n=105); Male (n=60)  *Demographics*  *Age*: M=45.6 (SD=14.2) years  *BMI*: M=25.2 (SD=5.0) kg/m^2^ |
| Vancamfort et al^4^ | 48 | SIMPAQ | - | BREQ-3 | - | *Country*: Uganda  *Diagnosis*: Psychotic disorder (n=48)  *Sex*: Female (n=24); Male (n=24)  *Demographics*  *Age*: M=33.3 (SD=9.6) years  *BMI*: M=21.8 (SD=2.9) kg/m^2^ |
| Vancampfort et al^5^ | 50 | SIMPAQ | - | BREQ-3 | *-* | *Country*: Uganda  *Male* (n=50)  *Diagnoses*: Alcohol use disorder  *Demographics*  *Age*: M=33.0 (SD=10.7) years  *BMI*: M=21.8 (SD=2.9) kg/m^2^ |
| Seymour et al (n=94)^6^  (some results unpublished) | 239 | 2PAQ | - | BREQ-3 | Medical screen; K6 | *Country*: Australia  *Sex:* Female (n=142); Male (n=97)  *Diagnoses*: Psychotic disorder (n=69); Affective disorder (n=131); Anxiety disorder (n=10); Other (n=11)  *Demographics*  *Age*: M=41.5 (SD=8.6) years  *BMI*: M=35.6 (SD=12.9) kg/m^2^ |
| Korman et al^7^ | 13 | SIMPAQ | SIMPAQ | BREQ-2 | Medical screen; BPRS | *Country*: Australia  *Sex:* Female (n=4); Male (n=9)  *Diagnoses*: Psychotic disorder (n=12); Affective disorder (n=1)  *Demographics*  *Age*: M=32.3 (SD=8.1) years  *BMI*: M=28.0 (SD=4.9) kg/m^2^ |
| Williams et al^8^ | 40 | IPAQ  Accelerometry | IPAQ_SF  Accelerometry | BREQ-2 | *-* | *Country*: England  *Sex:* Female (n=22); Male (n=18)  *Diagnoses*: Psychotic disorder (n=31); Affective disorder (n=6); missing (n=3)  *Demographics*  *Age*: M=43.3 (SD=8.2) years  *BMI*: M=32.4 (SD=6.6) kg/m^2^ |
| Korman et al^9^ | 42 | SIMPAQ  Accelerometry | SIMPAQ  Accelerometry | BREQ-2 | Medical screen; BPRS | *Country*: Australia  *Sex:* Female (n=9); Male (n=33)  *Diagnoses*: Psychotic disorder (n=39); Affective disorder (n=3); Substance use disorder (n=1)  *Demographics*  *Age*: M=33.3 (SD=9.4) yrs  *BMI*: M=29.3 (SD=6.5) kg/m^2^ |
| Chapman et al^10^ (results unpublished) | 69 | SIMPAQ  Accelerometry | SIMPAQ  Accelerometry | BREQ-3 | Medical screen; K6 | *Country*: Australia  *Sex:* Female (n=27); Male (n=42)  *Diagnoses*: Psychotic disorder (n=43); Affective disorder (n=18); Other (n=8)  *Demographics*  *Age*: M=35.9 (SD=10.5) years  *BMI*: M=31.9 (SD=7.9) kg/m^2^ |
| Korman et al (unpublished) | 49 | SIMPAQ  Accelerometry | SIMPAQ  Accelerometry | BREQ-3 | Medical screen; BPRS | *Country*: Australia  *Sex:* Female (n=13); Male (n=36)  *Diagnoses*: Psychotic disorder (n=46); Affective disorder (n=3)  *Demographics*  *Age*: M=32.8 (SD=10.4) years  *BMI*: M=32.2 (SD=8.2) kg/m^2^ |


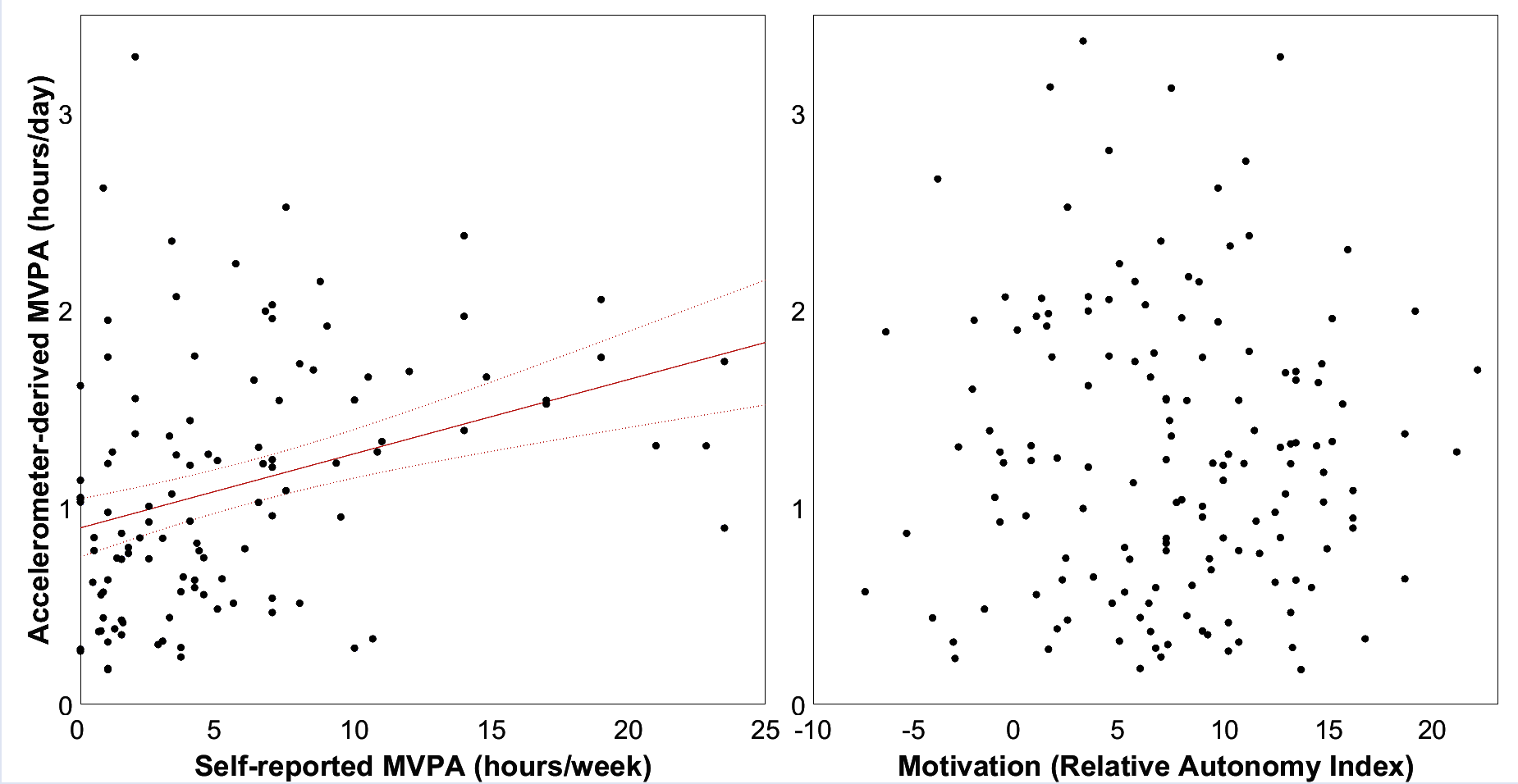


**Supplementary Figure 1:** Accelerometer-derived moderate-to-vigorous physical activity (MVPA; n=137) was correlated with self-reported MVPA (r=0.406, p<0.001; left panel), but not Relative Autonomy Index (p=0.44; right panel). The regression of accelerometer-derived MVPA against self-reported MVPA is plotted with the standard error of the mean (left).


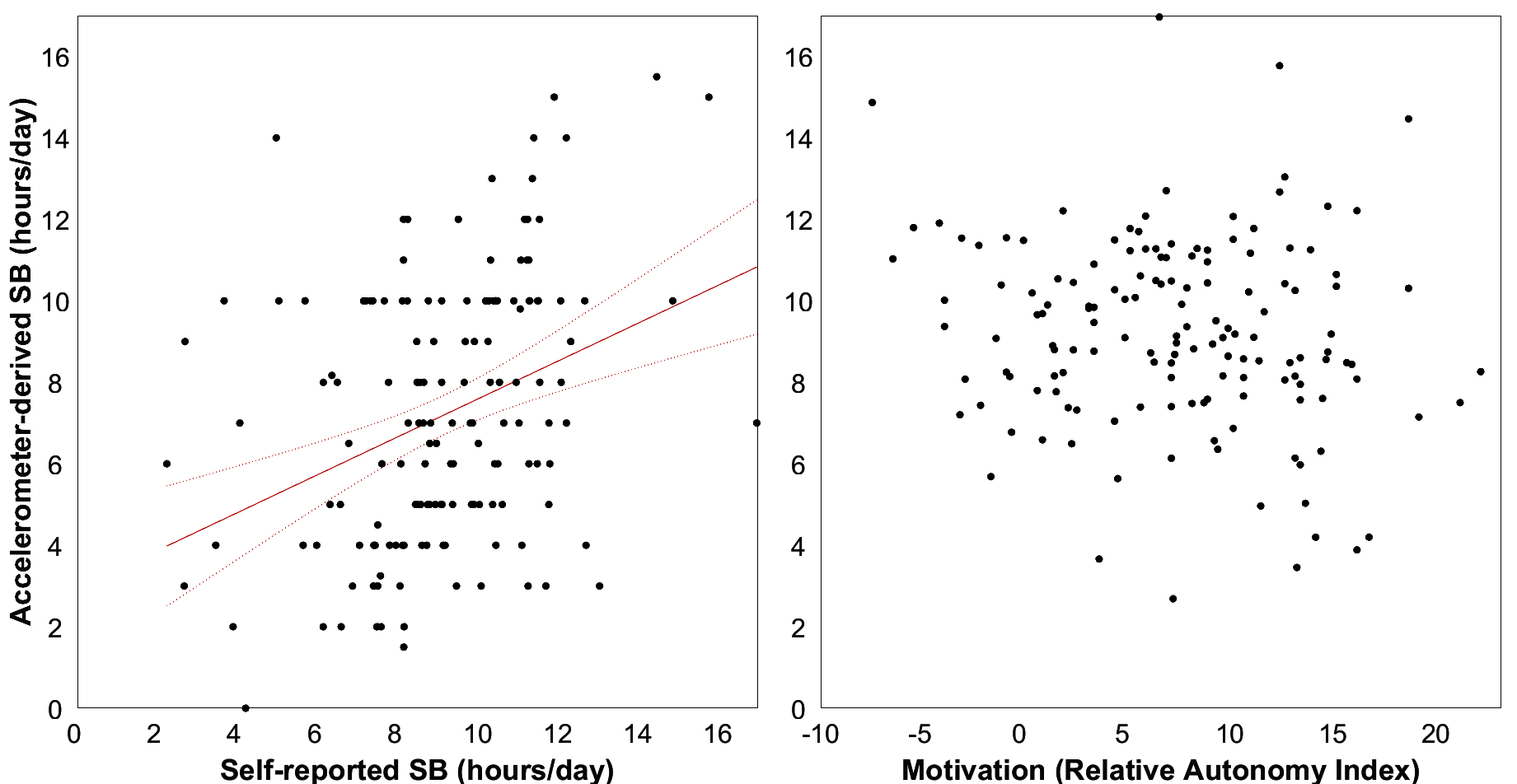


**Supplementary Figure 2:** Accelerometer-derived sedentary behaviour (SB; n=139) was correlated with self-reported SB (r=0.347, p<0.001; left panel), but not Relative Autonomy Index (p=0.09; right panel). The regression of accelerometer-derived SB against self-reported SB is plotted with the standard error of the mean (left).

1. Vancampfort D, De Hert M, Broderick J, et al. Is autonomous motivation the key to maintaining an active lifestyle in first‐episode psychosis? *Early Interv Psychiatry.* 2018;12(5):821-827.

2. Vancampfort D, Moens H, Madou T, et al. Autonomous motivation is associated with the maintenance stage of behaviour change in people with affective disorders. *Psychiatry Res.* 2016;240:267-271.

3. Vancampfort D, Madou T, Moens H, et al. Could autonomous motivation hold the key to successfully implementing lifestyle changes in affective disorders? A multicentre cross sectional study. *Psychiatry Res.* 2015;228(1):100-106.

4. Vancampfort D, De Hert M, Probst M, et al. Interest, competence, appearance, fitness and social relatedness as motives for physical activity in Ugandan outpatients with psychosis. *Ment Health Phys Act.* 2017;13:94-99.

5. Vancampfort D, Van Damme T, Probst M, et al. Motives for physical activity in the adoption and maintenance of physical activity in men with alcohol use disorders. *Psychiatry Res.* 2018;261:522-526.

6. Seymour J, Pratt G, Patterson S, et al. Changes in self-determined motivation for exercise in people with mental illness participating in a community-based exercise service in Australia. *Health Soc Care Comm.* 2021;30(5):e1611-e1624.

7. Korman NH, Shah S, Suetani S, et al. Evaluating the feasibility of a pilot exercise intervention implemented within a residential rehabilitation unit for people with severe mental illness: GO HEART:(Group Occupational Health Exercise and Rehabilitation Treatment). *Front Psychiatry.* 2018;9:343.

8. Williams J, Stubbs B, Richardson S, et al. ‘Walk this way’: results from a pilot randomised controlled trial of a health coaching intervention to reduce sedentary behaviour and increase physical activity in people with serious mental illness. *BMC psychiatry.* 2019;19(1):287.

9. Korman N, Fox H, Skinner T, et al. Feasibility and Acceptability of a Student-Led Lifestyle (Diet and Exercise) Intervention Within a Residential Rehabilitation Setting for People With Severe Mental Illness, GO HEART (Group Occupation, Health, Exercise And Rehabilitation Treatment). *Front Psychiatry.* 2020;11:319.

10. Chapman JJ, Suetani S, Siskind D, et al. Protocol for a randomised controlled trial of interventions to promote adoption and maintenance of physical activity in adults with mental illness. *BMJ open.* 2018;8(9):e023460.
